# Distinct Preflowering Drought Tolerance Strategies of *Sorghum bicolor* Genotype RTx430 Revealed by Subcellular Protein Profiling

**DOI:** 10.3390/ijms21249706

**Published:** 2020-12-19

**Authors:** Aaron J. Ogden, Shadan Abdali, Kristin M. Engbrecht, Mowei Zhou, Pubudu P. Handakumbura

**Affiliations:** 1 Earth and Biological Sciences Directorate, Pacific Northwest National Laboratories, Richland, WA 99354, USA; Aaron.Ogden@pnnl.gov (A.J.O.); Shadan.Abdali@pnnl.gov (S.A.); Kristin.Engbrecht@pnnl.gov (K.M.E.); 2 Environmental Molecular Sciences Laboratory, Pacific Northwest National Laboratory, Richland, WA 99354, USA; Mowei.Zhou@pnnl.gov

**Keywords:** drought stress, adaptive mechanisms, proteomics, sorghum, preflowering, postflowering, drought tolerance, FLOWERING LOCUS C, rubisco activase, abscisic acid, abiotic stress, reactive oxygen species

## Abstract

Drought is the largest stress affecting agricultural crops, resulting in substantial reductions in yield. Plant adaptation to water stress is a complex trait involving changes in hormone signaling, physiology, and morphology. Sorghum (*Sorghum bicolor* (L.) Moench) is a C4 cereal grass; it is an agricultural staple, and it is particularly drought-tolerant. To better understand drought adaptation strategies, we compared the cytosolic- and organelle-enriched protein profiles of leaves from two *Sorghum bicolor* genotypes, RTx430 and BTx642, with differing preflowering drought tolerances after 8 weeks of growth under water limitation in the field. In agreement with previous findings, we observed significant drought-induced changes in the abundance of multiple heat shock proteins and dehydrins in both genotypes. Interestingly, our data suggest a larger genotype-specific drought response in protein profiles of organelles, while cytosolic responses are largely similar between genotypes. Organelle-enriched proteins whose abundance significantly changed exclusively in the preflowering drought-tolerant genotype RTx430 upon drought stress suggest multiple mechanisms of drought tolerance. These include an RTx430-specific change in proteins associated with ABA metabolism and signal transduction, Rubisco activation, reactive oxygen species scavenging, flowering time regulation, and epicuticular wax production. We discuss the current understanding of these processes in relation to drought tolerance and their potential implications.

## 1. Introduction

Drought is the primary abiotic factors negatively affecting agriculture [1], causing tremendous losses in yield worldwide [2,3]. Current climate models predict rising seasonal temperatures and incidence of drought [2], and human population growth is expected to reach 9.7 billion by 2050 [4]. To prevent global food shortages, we must develop a better understanding of drought adaptation strategies in plants. In response to drought, general adaptation strategies include accelerating the transition from vegetative to reproductive (escape), increasing internal water content (avoidance), and to continue growth with decreased internal water content (tolerance) [5]. Each of these adaptation strategies require complex molecular responses to drought, including hormone signaling [6] and production of osmoprotectants [7], as well as developmental responses including stomatal closure, changes to leaf morphology, and altered root system architecture [8]. A common strategy to better understand these adaptation mechanisms is to compare plants with differing drought tolerances; this has been successful in multiple crop plants including wheat, rice, maize, and sorghum [9].

Sorghum (*Sorghum bicolor* (L.) Moench) (referred to as sorghum hereafter) is among the top five major cereal crops; it is used for food, biofuel production, and livestock feed [10]. Sorghum is a C4 grass, has a small and sequenced diploid genome, and is particularly drought-tolerant, making it an ideal candidate to identify novel drought adaptation strategies. Recent studies have identified multiple quantitative trait loci (QTLs) that control drought tolerance in sorghum at multiple life stages [11,12]. Multiple reports have identified molecular differences between sorghum genotypes differing in water use efficiency and drought tolerance. For example, increased expression of dehydrins and heat shock proteins as well as accumulation of specific sugars and sugar alcohols were identified as unique drought tolerance mechanisms in drought-tolerant sorghum Samsorg 17 compared to drought-susceptible sorghum Samsorg 40 [13]. Similarly, a comparison of transcript-level drought responses of sorghum genotypes IS22330 and IS20351 (drought-tolerant and -susceptible, respectively) suggests proteins involved in ROS scavenging, biosynthesis of secondary metabolites (e.g., phenylpropanoids, flavonoids), and a constitutively higher expression of cuticular wax biosynthesis genes are partly responsible for IS22330′s drought tolerance [9].

Comparisons of protein-level drought response using sorghum genotypes with differing drought tolerance identified the putative Rubisco chaperone HSP60 as changing uniquely to drought-tolerant cultivars [14]. Similarly, protein-level drought responses specific to the drought-tolerant sorghum line EL9 and not the drought-sensitive line Tabat included Rubisco large chain, a heat-shock protein, a glycine-rich RNA binding protein, and chloroplastic malate dehydrogenase [15]. Two sorghum genotypes with different drought tolerance, RTx430 and BTx642, are characterized as preflowering drought-tolerant, and postflowering drought-tolerant, respectively [16,17]. A large-scale RNA-Seq study found that preflowering drought stress caused similar transcriptional responses, but that the magnitude of changes in gene expression were different between genotypes [18]. Varoquaux et al. also found RTx430 genotype-specific transcriptional changes during preflowering drought stress, suggesting specific adaptation strategies, including upregulation of genes involved in shikimate pathway (relative to BTx642), increased glutathione-S transferase (GST) activity, and increased proline content [18]. Interestingly, preflowering drought induced inhibitors of ABA signaling in both sorghum genotypes, suggesting sorghum adapts to drought in part by preventing excessive ABA responses [18,19]. Recently, a comparison of histone modifications from drought-stressed RTx430 and BTx642 also revealed distinct histone posttranslational modification profiles between the two genotypes [20]. While most of the literature uses transcriptomics, experiments at both the protein and transcript levels suggest that photosynthesis and other organelle-localized processes are important for drought adaptation. Despite this trend, little is known about the protein-level response to drought in organelles.

The abundance of cytosolic proteins in whole-cell data-dependent proteomics experiments likely precludes identification of lower-abundance organelle proteins and their changes during water deficit. This study aimed to better understand RTx430′s unique drought tolerance by performing protein profiling of cytosolic- and organelle-enriched cellular compartments (CCs) in response to drought and comparing them with the preflowering drought-sensitive genotype BTx642. We extracted proteins from sorghum leaf tissues collected from a field study with well-controlled drought stress [18]. Our successful enrichment of the different CCs was confirmed by the far higher abundances of chloroplastic and histone proteins in organelle-enriched samples, and higher levels of ribosomal proteins in cytosolic-enriched samples. In response to drought, we observed many protein-level responses in both cellular compartments and genotypes that are consistent with previous literature findings. We show that the drought responses of organelle-enriched samples differs between RTx430 and BTx642, while the response of cytosolic protein profiles was similar between genotypes. Organelle-enriched proteins whose abundance significantly changed exclusively in RTx430 upon drought stress suggest multiple drought adaptation strategies. These include an RTx430-specific change in proteins associated with ABA metabolism and signal transduction, Rubisco activation, and flowering time regulation. We discuss the current understanding of these processes in relation to drought tolerance and their potential implications.

## 2. Results

### 2.1. Protein Profiles Are Enriched for Organelle and Cytoplasmic Compartments

**Figure 1:**
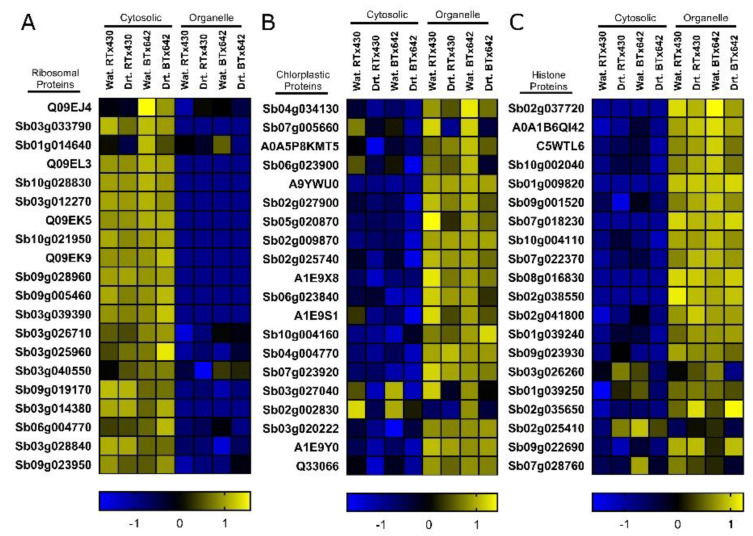
Evaluation of cytosolic and organelle enrichment. Ribosomal proteins abundant in the cytoplasm were more abundant in cytosolic-enriched samples (**A**), while photosynthesis-related chloroplastic (**B**) and nuclear histone proteins (**C**) were more abundant in organelle-enriched samples. Color and scale bars represent average *Z*-score transformed protein abundances of all biological replicates for each sample. Protein identifiers correspond to STRING or UniProt accessions. Wat., Watered; Drt., Drought.

The *S. bicolor* genotype RTx430 exhibits a unique preflowering drought tolerance compared to the preflowering drought sensitive genotype BTx642. To identify the potential mechanisms by which RTx430 uniquely adapts to preflowering water deficit, we compared the RTx430 and BTx642 organelle and cytoplasmic protein profiles after 8 weeks of drought stress preanthesis. To evaluate whether our procedure effectively enriched for organelle and cytoplasmic protein profiles, we first compared the abundance of histone, chloroplastic, and cytosolic ribosomal proteins independent of drought stress (Figure 1). In total, our analysis resulted in confident identification of 3683 and 4130 proteins in cytoplasmic- and organelle-enriched samples, respectively. A total of 3103 proteins were identified in both sample types (Supplemental Table S1). We observed cytosolic ribosomal proteins, including ribosomal L13, S16, and L20 were more abundant in cytosolic protein samples (Figure 1A). Conversely, chloroplastic and histone proteins were predominately more abundant in organelle-enriched samples (Figure 1B,C). These data suggest a successful enrichment for protein profiles representative of the different cellular compartments.

### 2.2. Organelle-Enriched Protein Profiles Comprise Genotype-Specific Drought Signatures

**Figure 2:**
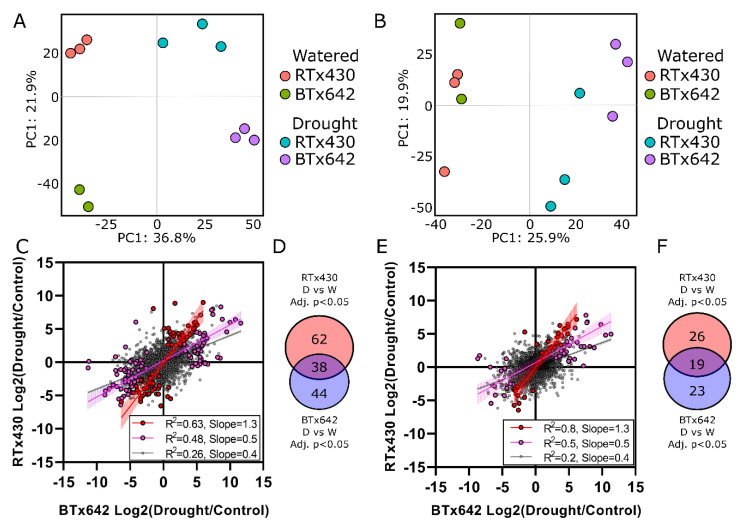
Principal component analysis of organelle- (**A**) and cytosolic-enriched protein profiles (**B**) in response to drought. Correlation between RTx430 and BTx642 drought responses in (**C**) organelle- and (**E**) cytoplasm-enriched samples. Venn diagram summary of proteins with adj. *p*-value < 0.05 in each genotype for (**D**) organelle- and (**F**) cytosolic-enriched samples. Purple data points are significantly changed in either genotype, while red points correspond to RTx430-specific changes (adj. *p*-value < 0.05). D, Drought; W, Watered; PC, Principal Component.

To evaluate the extent to which drought impacted the proteome of organelle- and cytoplasm- enriched samples, we first performed principal component analysis (PCA). For organelle-enriched samples, principal component (PC) 1 comprises 36.8% of data variation caused by drought (Figure 2A). Similarly, PC2 comprises 21.9% of data variation and is caused by differences between genotypes. Similarly, for cytosolic-enriched samples, 25.9% of data variation is explained in PC1 and appears to be caused by drought (Figure 2B). Unlike organelle-enriched samples, cytosolic-enriched samples do not appear to separate by genotype on PC2. These data suggest that drought impacts both organelle- and cytoplasm-enriched samples, and that the drought response of organelles may be more genotype-specific compared with the cytosol. A statistical comparison of organelle-enriched samples identified 100 and 99 drought-responsive DEPs in RTx430 and BTx642, respectively, with an overlap of 38 (Figure 2D). Similarly, a statistical test of cytosolic-enriched samples using limma [21,22] identified 45 and 42 drought-responsive differentially expressed proteins (DEPs, adj. *p*-value < 0.05) in RTx430 and BTx642, respectively, with an overlap of 19 (Figure 2F). Previous reports suggest that the drought stress responses of these genotypes are largely similar, but that the magnitude of RTx430′s response is larger than BTx642′s [18]. Consistent with this observation, the cytosolic- and organelle-enriched drought responses showed positive correlation (*r*^2^ = 0.5, slope = 0.5, Figure 2C,E). However, the DEPs exclusive to RTx430′s stress response showed a much larger change during drought (Figure 2C,E).

### 2.3. Protein Profiles in Response to Drought Support Previous Findings

To evaluate whether our protein profiles accurately captured a drought response, we first performed Gene Ontology (GO) enrichment [23] using DEPs that increase significantly >2-fold in response to drought (limma adj. *p*-value < 0.05). Drought-relevant GO terms, including response to heat (GO:0009408), response to osmotic stress (GO:0006970), and response to abiotic stimulus (GO:0009628) were significantly enriched among upregulated DEPs (Fisher’s Exact, Bonferroni adj. *p*-value < 0.05) (Supplemental Table S1). Proteins belonging to these GO terms include multiple small heat shock proteins (SHSPs) (e.g., Sb03G003530 and Sb01G040030) [24], as well as the water stress-induced dehydrin C5YX70 all of which were significantly >2-fold more abundant in response to drought in both genotypes (adj. *p*-value < 0.05). We also found that our data support previous findings. For example, the SHSPs Sb06G017850, Sb03G006870, Sb07G028370, Sb03G003530, and Sb04G035130 were significantly >2-fold more abundant in response to drought in cytosolic-enriched RTx430 and BTx642 samples, and were similarly upregulated in the study described in Johnson et al. 2014 [25]. Similarly, the dehydrin protein Sb09G018420, thioredoxin protein Sb03G036980, and glucose-1-phosphate adenylyltransferase Sb09G029610 were significantly >2-fold more abundant in our experiments and elsewhere [25]. We also observed a significant >2-fold increase in the AAA domain-containing protein Sb03G001130, and ribosomal L16 protein Sb01G036330 in cytosolic-enriched RTx430 and BTx642 during drought stress, consistent with observations in Abdel-Ghany et al., 2020 [26]. A cross reference of our observations with literature findings can be found in Supplemental Table S1. These findings suggest our experiment corroborates previous research findings and accurately captured protein profiles representative of sorghum’s drought response.

### 2.4. Genotype-Specific Drought Responses Involved in Flowering Time Control, Starch Biosynthesis, and Rubisco Activation May Explain RTx430′s Drought Tolerance

**Figure 3:**
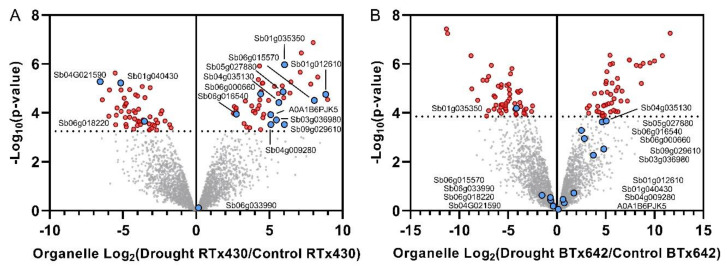
Comparison of genotype-specific drought responses in organelle-enriched samples of RTx430 (**A**) and BTx642 (**B**). Red datapoints passed a limma adjusted *p*-value threshold of 0.05. Pink circles indicate proteins that changed significantly only in RTx430 (limma adj. *p*-value < 0.05). Protein names correspond to STRING identifiers, or when unavailable, UniProt accessions.

A comparison of significant drought-induced protein abundance changes in RTx430 with BTx642 identified multiple proteins that changed exclusively in RTx430 (Table 1). For example, the likely CDC73/PHP protein Sb01G012610 was significantly >100-fold more abundant in RTx430 organelle-enriched samples in response to drought and did not change significantly in BTx642 (Figure 3). The nearest *Arabidopsis* ortholog of Sb01G012610 is AtCDC73/PHP (At3G22590), and loss of AtCDC73 function caused reduced H3K4me3 histone methylation patterns at the FLOWERING LOCUS C (FLC), misregulation of FLC expression, and an accelerated transition from vegetative to reproductive growth [27,28]. These data suggest the RTx430-specific increase in CDC73-like Sb01G012610 may function similarly to suppress FLC-mediated transition from vegetative to reproductive growth during drought.

We also observed a significant 65-fold drought-responsive increase in the ADP-Glucose pyrophosphorylase (Sb09G029610) exclusively in organelle-enriched RTx430 samples (Figure 3). The closest *Arabidopsis* ortholog of Sb09G029610 is the glucose-APL2 (At1G27680), which catalyzes the first rate-limiting step in starch synthesis [29]. Starch biosynthesis plays an active role in drought tolerance in *Z. mays*, and loss of function in similar ADP-glucose pyrophosphorylases (e.g., SH2) leads to soluble sugar starvation and reduced leaf growth rates [30]. We observed a significant 61-fold drought-responsive increase in the rubisco activase (RCA) protein Sb05G027880 exclusively in RTx430. In C3 and C4 grasses, RCA plays a critical role in maintaining photosynthesis during heat and drought stress [31]. Taken together, these data suggest that altered starch metabolism, Rubisco activation, and suppression of the transition from vegetative to reproductive growth may be preflowering drought adaptation strategies specific to *S. bicolor* RTx430.

### 2.5. Genotype-Specific Drought Responses Involved in Abscisic Acid Signaling May Explain RTx430′s Drought Tolerance

We observed a significant 34-fold increase in the likely abscisic acid receptor PYL9 Sb04G009280 exclusively in organelle-enriched RTx430 samples (Figure 3). Interestingly, drought was shown to decrease PYL9 *Sorghum bicolor* L. Moench [32] (annotated as PYL8 in Dalal et al. 2014), and slightly downregulated in *S. bicolor* BTx642 in response to saline–alkali stress [33] (annotated as PYL6 in Ma et al. 2019). Nearest orthologs of Sb04G009280 are PYL9 genes (*Z. mays* Zm00001d016105 and *Arabidopsis* At1G01360 [34]), and *Z. mays* PYL9 overexpression lines were more resistant to drought [35,36]. We also observed a significant 11-fold decrease in the zeaxanthin epoxidase (ZEP, Sb06G018220) exclusively in organelle-enriched RTx430 during drought. Zeaxanthin epoxidase catalyzes the first step in ABA biosynthesis and plays a critical role in xanthophyll production [37] (Figure 3). In model grasses, decreased ABA production correlated with drought tolerance during vegetative [57] and reproductive growth [58]. We observed a significant 6.7-fold increase in the likely abscisic acid stress-ripening (ASR) protein Sb06G016540 exclusively in RTx430 organelle-enriched samples during drought. The Sb06G016540 protein is orthologous to *Z. mays* ASR3 and confers drought tolerance when expressed in *Arabidopsis* [38] and tobacco [39]. These data suggest that part of RTx430′s preflowering drought tolerance includes prevention of an excessive stress-induced ABA response by decreasing ABA production, while increasing PYL9 abundance may function to maintain sensitivity to ABA and stomatal control.

### 2.6. Other RTx430-Specific Preflowering Drought Responses Include Proteins Involved in ROS Scavenging, HSPs, Epicuticular Wax Production, and Phospholipid Metabolism

We observed a significant 65-fold increase in the superoxide dismutase (SOD) protein Sb01G035350 in organelle-enriched RTx430 in response to preflowering drought (Figure 3). Interestingly, the same protein was significantly 18-fold decreased in organelle-enriched BTx642 during drought. Orthologs of SOD in *Arabidopsis* and rice include AtCSD1 (At1g08830) and SODCC1 (Os03g22810), and have been shown to play a role in adaptation to high light and drought [40]. Another ROS scavenging enzyme, Ferredoxin (Sb06G015570), was significantly >100-fold more abundant exclusively in RTx430 organelles during drought. Ferredoxin was also upregulated in the drought-tolerant sorghum line SC56 compared with the drought-sensitive line Tx7000 [41]; it confers heat stress tolerance in *Arabidopsis* [42] and salt tolerance in rice [43]. We also observed a significant 44-fold increase in the thioredoxin protein Sb03G036980 (NRX1) exclusively in RTx430 organelles during drought. The NRX1 protein was previously shown to be drought-inducible in sorghum [14] and orthologous proteins (e.g., *Arabidopsis* AtNRX1 (At1G60420) and *Z. mays* Zm00001d012591) have been characterized. The *Arabidopsis* AtNRX1 was shown to guard sensitive enzymes from oxidative stress, and knockouts had reduced catalase activity and increased sensitivity to oxidative stress [44].

Another oxidative stress relevant protein, the sHSP Sb04G035130 was significantly 45-fold increased exclusively in organelle-enriched RTx430 samples (Figure 3). Sequence homology suggests Sb04G035130 is orthologous to HSP17.4B (*Arabidopsis* ortholog At1G54050 and *Z. mays* ortholog Zm00001d018298), which was shown to be involved in oxidative stress adaptation [45]. Similarly, we observed a significant 21-fold increase in the likely HSP90 protein C5YBL4 exclusively in drought stressed organelle-enriched RTx430 samples. The orthologs of Sb06G000660 are HSP90 proteins in *Arabidopsis* and *Z. mays* (At5g52640 and Zm00001d024903, respectively) and it was shown that AtHSP90 partly controls stomatal ontogenesis and heat stress adaptation by interacting with YODA to transduce the heat stress response, including phosphorylation of MPK6 and SPCH [46].

We observed a significant RTx430-specific 34-fold increase in 3-ketoacyl-CoA synthase (UniProt identifier A0A1B6PJK5), which catalyzes the first step in very-long-chain fatty acids (VLCFAs) necessary for epicuticular wax production [47]. Epicuticular wax is important for drought tolerance in multiple species [48], and its abundance in grass crops correlated with grain yield under drought stress [49], including sorghum [50,51]. Multiple drought-relevant proteins became significantly less abundant exclusively in organelle-enriched RTx430 during water deficit. For example, we observed a significant 43-fold reduction in the likely phospholipase A2 protein Sb01G040430 (PLA2) in organelle-enriched RTx430 samples during drought (Figure 3). Phospholipases hydrolyze membrane phospholipids, and they play a role in maintaining membrane integrity as well as production of fatty acid precursors of drought-relevant signaling molecules [52]. Mutant *Arabidopsis* plants lacking PLA2 show delayed stomata opening, and are increased during drought stress in the wheat [53]. We also observed a significant 93-fold decrease in the HMA domain-containing protein Sb04G021590 exclusively in RTx430 organelle-enriched samples during drought. Orthologous proteins in *Z. mays* and *Arabidopsis* (At3G56240 and Zm00001d016691, respectively) encode a copper chaperone CCH [54,55] important in maintaining electron transport and photosynthesis [56], and are downregulated during drought at the transcript level in *Sorghum bicolor* [9].

## 3. Discussion

Drought is the primary stress causing crop yield loss. Comparing closely related genotypes with differing drought tolerance has proved to be a successful way to identify plant adaptation strategies to water deficit stress. Two *Sorghum bicolor* genotypes, RTx430 and BTx642, are characterized as preflowering drought-tolerant and -sensitive, respectively. Multiple reports have examined the differences between each genotype in response to water limitation but have relied on whole-organ and transcript-level measurements. To further understand RTx430′s unique drought tolerance, we report here a close examination of the protein-level drought response in organelle- and cytosolic-enriched cellular compartments (CCs). Our analysis identified >3000 proteins in each CC that are enriched for proteins characteristic of each compartment, indicating a successful capture of organelle- and cytosolic-protein profiles. Our data set supports previous findings, including upregulation of multiple drought-responsive proteins previously characterized in the literature. Further examination of our data suggests that organelle-enriched protein profiles respond to drought in a genotype-specific manner more than cytosolic-enriched proteins and may shed light on water stress adaptation strategies unique to RTx430.

For example, drought caused an RTx430-specific increase in Sb01G012610 in organelle-enriched samples. Sequence homology suggests Sb01G012610 is a nuclear-localized PLANT HOMOLOGOUS TO PARAFIBROMIN (PHP/CDC73) protein, which has been characterized best in Arabidopsis as a component of the Paf1c complex [27]. The Paf1c complex is involved in regulation of transcription by altering histone methylation status and RNA processing [59,60,61]. Evidence suggests that Paf1c participates in controlling the transition from vegetative to reproductive growth via histone methylation at the FLOWERING LOCUS C (FLC) locus. The FLC is a well-characterized repressor of flowering, and it was recently shown that CDC73 associates with and modifies the histone methylation status of chromatin at the FLC locus, resulting in increased FLC expression [27,28]. Mutants lacking CDC73 exhibited reduced FLC expression and accelerated flowering times [27]. Differences between RTx430 and BTx642 histone methylation profiles during drought stress were recently reported [20], and the RTx430-specific increase in the CDC73 ortholog Sb01G012610 during drought observed here suggests a possible mechanism by which RTx430 suppresses flowering during water limitation and remains in a vegetative growth phase.

Drought tolerance requires maintaining photosynthetic rates despite decreasing cellular water availability, a process carried out in part by the Rubisco chaperone Rubisco Activase (RCA). RCA activates Rubisco by catalyzing the ATP-dependent removal of inhibitory sugar phosphates bound to RuBisCo [62], and RCA’s activity is sensitive to heat, chloroplast redox status, and ATP/ADP ratios [31]. *Arabidopsis* mutants with decreased RCA activity exhibited decreased growth and photosynthetic rates [63], and RCA expression is upregulated in drought-tolerant rice genotypes compared with drought-sensitive lines [64]. Furthermore, RCA protein levels are increased by heat stress in wheat [65]. Reviewed by Feller 2016, increased thermal stability of RCA at higher temperatures correlated with increased temperature optimum of photosynthesis, and consequently, RCA is often a target for breeding drought-tolerant crops [66]. In sorghum, rubisco activase gene expression is downregulated under heat stress, particularly for drought-sensitive lines [67]. While Rubisco and RCA activity was not measured here, the observation that RCA increased exclusively in *S. bicolor* RTx430 suggests that Rubisco maintenance contributes to RTx430′s preflowering drought adaptation strategy.

Starch biosynthesis occurs during photoperiods and is catabolized into soluble sugars that support respiration and growth at night. Drought typically increases starch content in leaves, and soluble sugars act as a carbon source, signal molecule, and osmolyte [68]. We also observed an RTx430-specific increase in the starch biosynthesis protein ADP-glucose pyrophosphorylase Sb09G029610 (AtAPL2 (At1g27680) and ZmAPL2 (Zm00001d039131) ortholog) during drought. While drought-induced changes in APL transcript or protein abundance is plant-specific (reviewed by Thalman et al., 2017 [68]), mutants impaired in starch metabolism often show impaired growth and drought tolerance [69]. In other grass crops like maize, APL2 mutants showed decreased drought tolerance, drought recovery [30], and growth. Our findings suggest that RTx430 adapts to preflowering drought stress in part by increasing starch biosynthesis.

We also observed RTx430-specific changes in proteins involved in ABA metabolism. ABA is a well-characterized stress hormone typically synthesized de novo in response to drought [19,70]. The first step in ABA biosynthesis is the epoxidation of zeaxanthin to antheraxanthin and eventually violaxanthin by zeaxanthin epoxidase (ZEP) [71]. Multiple studies have shown that drought-tolerant grasses accumulate less ABA during drought at vegetative and reproductive stages [57,58], and lower ABA in foliar tissues is often used as selection criteria for breeding drought-tolerant crops like maize [72]. It was shown in *Arabidopsis* that ZEP protein is predominantly chloroplastically localized and undergoes degradation upon drought stress [73,74], but Schwarz et al. report no significant change in ABA content, and posit that the decrease in ZEP functions to prevent zeaxanthin consumption, leaving additional zeaxanthin for production of photoprotective xanthophylls. Because ABA signaling and crosstalk is complex, and no measurements of ABA was made in this study, it remains to be tested whether the observed decrease in ZEP during preflowering drought reduced ABA levels in *S. bicolor* RTx430 leaves. However, our data suggests that decreased ZEP abundance in RTx430 but not BTx642 may be a possible adaptation strategy wherein RTx430 reduces ABA production and prevents an excessive stress response.

The primary receptors responsible for ABA perception and signal transduction are the PYROBACTIN RESISTANCE 1-Like (PYL) family proteins [75,76]. In guard cells, ABA binding to PYL results in inhibition of PP2C phosphatases and subsequent activation of SnRK2 kinases that leads to stomatal closure and decreased transpiration [77]. The PYL receptors are transcriptionally downregulated in drought-sensitive *S. bicolor* lines [32,33] and rice [78] but drought-inducible in *Z. mays* [36] and ABA-inducible in *Arabidopsis* [75,76]. Overexpression of PYL9 in *Arabidopsis* [35], rice [78], and maize [36] resulted in increased drought tolerance or ABA sensitivity. Our observation that *S. bicolor* PYL9 (Sb04G009280) significantly increased in genotype RTx430 and not in BTx642 suggests that ABA receptors are involved in RTx430′s preflowering drought tolerance. The only likely SnRK2 [32] identified here, SB06G033990, did not change significantly in either genotype or CC, and no likely PP2C’s were detected. It may be that the increased PYL observed here functions to maintain sensitivity to decreased ABA caused by lower ZEP protein abundance, allowing proper stomatal closure.

We also observed an RTx430-specific increase in the likely ASR (abscisic acid-, stress-, and ripening-induced) protein during preflowering drought. The *Z. mays* ASR (ZmASR3) was found to confer additional drought tolerance and longer roots when overexpressed in *Arabidopsis* [38]. The improved drought tolerance of *Arabidopsis* ZmASR3 overexpressing lines was attributed in part to a decreased ROS by increasing superoxide dismutase and catalase activity in leaves [38]. Similarly, other ASR orthologs in wheat (TaASR1) [39], tomato (ASR1) [79], lily (LLA23) [80], rice (OsAsr1) [81], and plantain (MpAsr) [82] conferred additional drought tolerance when overexpressed in tobacco, *Arabidopsis*, rice, and *Arabidopsis*, respectively. The *S. bicolor* RTx430-specific increase in the likely ASR (Sb06G016540) observed here suggests additional ASR production contributes to RTx430′s preflowering drought tolerance.

Epicuticular waxes are a complex mixture of lipids composed mostly of VLCFAs, and their production is critical for drought tolerance and photoprotection under high light in multiple species including maize, wheat, and soybean [48]. The first step in VLCFA biosynthesis is catalyzed by β-ketoacyl CoA synthase (KCS), and mutants lacking KCS function exhibit decreased drought tolerance in multiple species including rice [47,83] and sorghum [50,51,84]. The observation here that RTx430 exclusively increased KCS during drought suggests epicuticular wax production contributes to RTx430′s unique drought tolerance. Other proteins identified here that are known to increase drought tolerance include the ferredoxins. Ferredoxins are important in ROS scavenging, and their overexpression confers increased drought tolerance in multiple systems [42,43]. Our observations suggest RTx430′s unique drought tolerance may involve producing additional ROS scavenging ferredoxins.

To our knowledge, this study constitutes the first comprehensive protein profiling of sorghum subcellular compartments in response to drought. Consistent with previous observations, our findings suggest that while RTx430 and BTx642 respond similarly to drought, the magnitude of RTx430′s response is greater than BTx642 and these differences may be responsible for RTx430′s preflowering drought tolerance. Our data also suggest that genotype-specific changes in organelle protein profiles may be greater compared with the cytoplasm. The proteins identified as changing only in organelle-enriched RTx430 samples during drought implicate multiple biological processes in RTx430′s unique preflowering drought tolerance and provides multiple avenues for breeding crops with improved drought tolerance.

## 4. Materials and Methods

### 4.1. Plant Growth and Drought Stress

Plants used in this study were grown and collected by Xu et al., 2018 [85]. Briefly, two sorghum (*S. bicolor* (L.) Moench) genotypes, RTx430 and BTx642, were grown in Parlier, CA in a randomized block design. The average monthly rainfall at the field site during the growth season is approximately zero, and soils are characterized as sandy loam with silky substratum. All watering was accurately and uniformly controlled via drip lines at the furrow of each plant. Watered control plants received consistent watering at 80% of calculated evapotranspiration, as measured by an onsite weather station ~1 km from the field site. Preflowering drought was imposed by halting irrigation upon seedling emergence until sample collection 8 weeks later. Drought stressed plants therefore received no additional water for 8 weeks upon seedling emergence from the soil, while stress-free watered plants received consistent drip line irrigation based on local evapotranspiration measurements. Drip line emitters were spaced 0.3 m apart with an output of 2 L/h, and watering was conducted once a week. Less than 1 mm of rainfall was recorded during the growth period at the experiment site. Control and drought stressed sorghum leaves were manually excised at 8 weeks postemergence and frozen in liquid nitrogen prior to protein isolation. Samples used in this study included 3 biological replicates of *S. bicolor* RTx430 drought and control, as well as 2 control and 3 drought biological replicates of *S. bicolor* BTx642. Each biological replicate is a pool of 10 individuals from each treatment group, collected from the same row of plants, at the same date and time. The percent of plant-available soil moisture was estimated using soil matric potential sensors (Watermark sensors, Irrometer Corp., Riverside, CA, USA) placed 15 cm beneath the soil and centered within plant rows. The degree of water stress was measured using the crop water stress index (CWSI) for both drought and watered treatments [85]. Additional detailed metrics used to evaluate the extent of drought as well as the resulting plant phenotype can be found in Xu et al. 2018 [85].

### 4.2. Organelle and Cytosolic Enrichment

Organelle and cytosolic protein fractions were obtained using a modified protocol adapted from O’Green et al. 2011 [86] and our histone purification protocol [20]. Briefly, leaves were homogenized by bead beating at −80 °C, and resuspended in buffer EB1 (0.44 M Sucrose, 10 mM Tris-HCl pH 8.0, 5 mM DTT) containing protease inhibitor cocktail (Sigma 5892791001, St. Louis, MO, USA). Plant debris was filtered through mesh 100 cloth (Sigma F6801, St. Louis, MO, USA) and organelles were pelleted by centrifugation at 3000× *g* for 10 min at 4 °C. The supernatant containing the cytosolic fraction was collected and frozen at −80 °C. The organelle pellet was resuspended in cold buffer EB2 (0.25 M sucrose, 10 mM MgCl2, 10 mM Tris-HCl pH 8.0, 1% Triton X100, 5 mM DTT) containing protease inhibitor cocktail and incubated on ice for 10 min with gentle mixing. Organelles were pelleted by centrifugation at 2100× *g* for 15 min at 4 °C, and supernatant was decanted. Pellet was then washed twice by resuspension and centrifugation in buffer EB2B (0.25 M sucrose, 10 mM MgCl_2_, 10 mM Tris-HCl pH 8.0, 5 mM DTT) at 2100× *g* for 15 min at 4 °C. Nuclei were lysed by resuspension in 200 µL of high salt nuclear lysis buffer (400 mM NaCl, 10 mM Tris-HCl pH 8, 1% Triton x100, 20 mM EDTA). Protein was frozen at −80 °C until quantification and digestion.

### 4.3. Protein Digestion

To remove plant metabolites that may interfere with downstream processes, protein from cytosolic-enriched samples were precipitated by MPLEx [87] and resuspended in 50 mM ammonium bicarbonate containing 8 M urea. The protein yield of organelle-enriched samples was relatively low and contained fewer contaminating metabolites; therefore, it was immediately resuspended in 50 mM ammonium bicarbonate with 8 M urea to prevent protein loss during MPLEx precipitation. Protein from all samples was then quantified by bicinchoninic acid assay (BCA), and disulfide bonds were reduced by addition of dithiothreitol to the final concentration of 10 mM and incubation at 60 °C for 30 min with shaking. Cysteine residues were alkylated by addition of iodoacetamide to a final concentration of 40 mM and incubation with shaking at 37 °C for 3 h. Protein was trypsin digested at a 50:1 (*w*/*w*) protein to trypsin ratio for 3 h at 37 °C with shaking at 800 rpm. Peptides were then desalted using C18 SPE Phenomenex (Torrance, CA, USA), dried down, and resuspended in water and stored at −80 °C.

### 4.4. LC-MS/MS Analysis

Peptides were separated using a Waters nanoAcquity LC (Waters Corporation, Milford, MA, USA) with binary solvent mobile phase A (0.1% (*v*/*v*) formic acid in water) and mobile phase B (0.1% FA (*v*/*v*) in water). 0.5 µg peptides were loaded onto a C18 reversed phase trap column (5 cm length, 100 μm i.d, 3 μm particle, 300 Å pore size, Phenomenex, Torrance, CA, USA) and washed with mobile phase A for 10 min at 3 µL/min. Then, the peptides are loaded onto a C18 analytical column (70 cm, 75 μm i.d, same material as the trap column). The analytical gradient was ramped from 1% to 45% mobile phase B over 100 min at 300 nL/min. Eluting peptides were injected in-line into a Q Exactive Orbitrap mass spectrometer (Thermo Fisher Scientific, Bremen, Germany) via electrospray ionization with data-dependent acquisition. The scan range was limited to 300–1800 m/z in positive mode, with resolution of 70,000. MS2 spectra were acquired with resolution of 17,500, scan range 200–2000 m/z, and 30 s dynamic exclusion. Raw spectra and associated data were submitted to the Mass Spectrometry Interactive Virtual Environment (MassIVE; [https://massive.ucsd.edu]) accession number MSV000086332.

### 4.5. Protein Search and Data Analysis

Peptide sequence identification was carried out using MaxQuant [88] Andromeda [89]. Search parameters included variable methionine oxidation, N-terminal acetylation, and fixed carbamidomethylation. Proteins were identified with 1% false discovery rate (FDR) using a comprehensive *S. bicolor* protein database from Uniprot [90] (downloaded September 2020) and iBAQ normalized. Data were then further processed in R using the methods described by Zhang et al., 2018 [22]. Data were prefiltered to remove contaminants and proteins identified by only a single unique peptide. Only proteins identified in all biological replicates of at least one treatment group (e.g., all RTx430 control, or all RTx430 drought, etc.) were included in subsequent analyses. Data were subjected to variance stabilized normalization (VSN, Supplementary Figure S1), and missingness was evaluated. Proteins with missing values were nonrandom and predominately occurred for proteins near the limit of detection (Supplementary Figures S2 and S3). We therefore chose to impute missing values by random sampling from a left-shifted Gaussian distribution (shift = 1.8, scale = 0.3) (Supplementary Figures S2 and S3). Differentially expressed proteins (DEPs) were identified by applying an empirical Bayes linear model using limma [21,91]. Statistical analysis also included principal component analysis (PCA) of cytoplasmic- and organelle-enriched samples. For comparison of cytoplasmic- and organelle-enriched samples for Figure 1, data were *Z*-score transformed in Perseus [92] (χ-µ/σ, where χ = observed value, µ = mean of all samples, σ = standard deviation of all samples). A comprehensive list of proteins identified here, their relative abundances, statistics, orthologous proteins, ontology enrichment, and alternative identifiers from UniProt, STRING, and Ensembl Plants can be found in Supplemental Table S1.

## Figures and Tables

**Table 1:** RTx430-specific drought responsive proteins.

Identifier	Protein	Abbr.	Ens. Ortholog	Function	Ref.
Sb01g012610	Plant Homologous to Parafribomin	CDC73/PHP	At3g22590, Zm00001d013597, Os03g0664700	Histone methylation, FLC-regulation	[27,28]
Sb09g029610	ADP-Glc pyrophosphorylase	APL2	At1G27680, Zm00001d039131, Os05g0580000	Starch biosynthesis	[29,30]
Sb05g027880	Rubisco activase alpha isoform	RCA	At2G39730, Zm00001d048592,	Photosynthesis	[31]
Sb04g009280	Pyrobactin Resistance 1-like	PYL9	At1G01360, Zm00001d016105, Os02g0255500	ABA Signal Transduction	[32,33,34,35,36]
Sb06g018220	Zeaxanthin epoxidase	ZEP	At5G67030, Os04g0448900	ABA metabolism	[37]
Sb06g016540	Abscisic Acid Stress Ripening	ASR2	Zm00001d003712, Os04g0423400	ABA metabolism	[38,39]
Sb01g035350	Superoxide dismutase	SOD	At1G08830, Zm00001d047479, Os03g0351500	ROS Scavenging	[40]
Sb06g015570	Ferredoxin	Fd	At2G27510, Zm00001d003797, Os04g0412200	ROS Scavenging	[41,42,43]
Sb03g036980	NRX1-like Thioredoxin domain-containing	NRX	Zm00001d012591, Os01g0794400	Oxidative Stress	[14,44]
Sb04g035130	Small heatshock protein 17	sHSP17	At1G54050, Zm00001d018298, Os02g0782500	Protein Folding	[45]
Sb06g000660	Heatshock protein 90	HSP90	At5G52640, Zm00001d024903, Os04g0107900	Protein Folding	[46]
A0A1B6PJK5	3-ketoacyl-CoA synthase	KCS	At1G25450, Os04g0116800	Epicuticular Wax Production	[47,48,49,50,51]
Sb01g040430	Phospholipase A2	PLA2	Zm00001d028505	Fatty acid and membrane integrity	[52,53]
Sb04G021590	HMA domain-containing protein	HMA	At3G56240, Zm00001d016691	Copper Ion Homeostasis	[9,54,55,56]
Sb06g033990	Sucrose non-fermenting 1-related prot. Kinase 2	SnRK2	At1G60940, Zm00001d026690, Os04g0691100	ABA Signal Transduction	[32]

Identifiers consist of STRING and UniProt accessions. Ensemble Orthologs, when available, are reported for *Arabidopsis thaliana*, *Zea mays*, and *Oryza sativa*. Ens., Ensemble; Prot., Protein; Ref., Reference; Abbr., Abbreviated Name; ADP, Adenosine diphosphate; Glc, Glucose.

## Supplementary Materials

Supplementary materials can be found at https://www.mdpi.com/1422-0067/21/24/9706/s1. Figure S1: Data normalization and goodness of fit; Figure S2: Missingness caused by low abundance proteins in organelle-enriched samples; Figure S3: Missingness caused by low abundance proteins in cytosolic-enriched samples; Table S1: Main data analysis table containing the normalized protein abundance values, relevant statistics, fold-changes, GO enrichment.

## Abbreviations

DEP	Differentially Expressed Proteins
ABA	Abscisic Acid
GO	Gene Ontology
FLC	FLOWERING LOCUS C
RCA	Rubisco Activase
ASR	Abscisic Acid Stress-Ripening
sHSP	Small Heat Shock Protein
ZEP	Zeaxanthin Epoxidase
SOD	Superoxide Dismutase
