# Supplementary material for: Distinct Preflowering Drought Tolerance Strategies of Sorghum bicolor Genotype RTx430 Revealed by Subcellular Protein Profiling
Source: Int J Mol Sci. 2020 Dec 19;21(24):9706. doi: 10.3390/ijms21249706 (PMC7767018; doi:10.3390/ijms21249706)
Supplement: Supplementary file 1 [file ijms-21-09706-s001.zip › ijms-995022-SI.pdf]

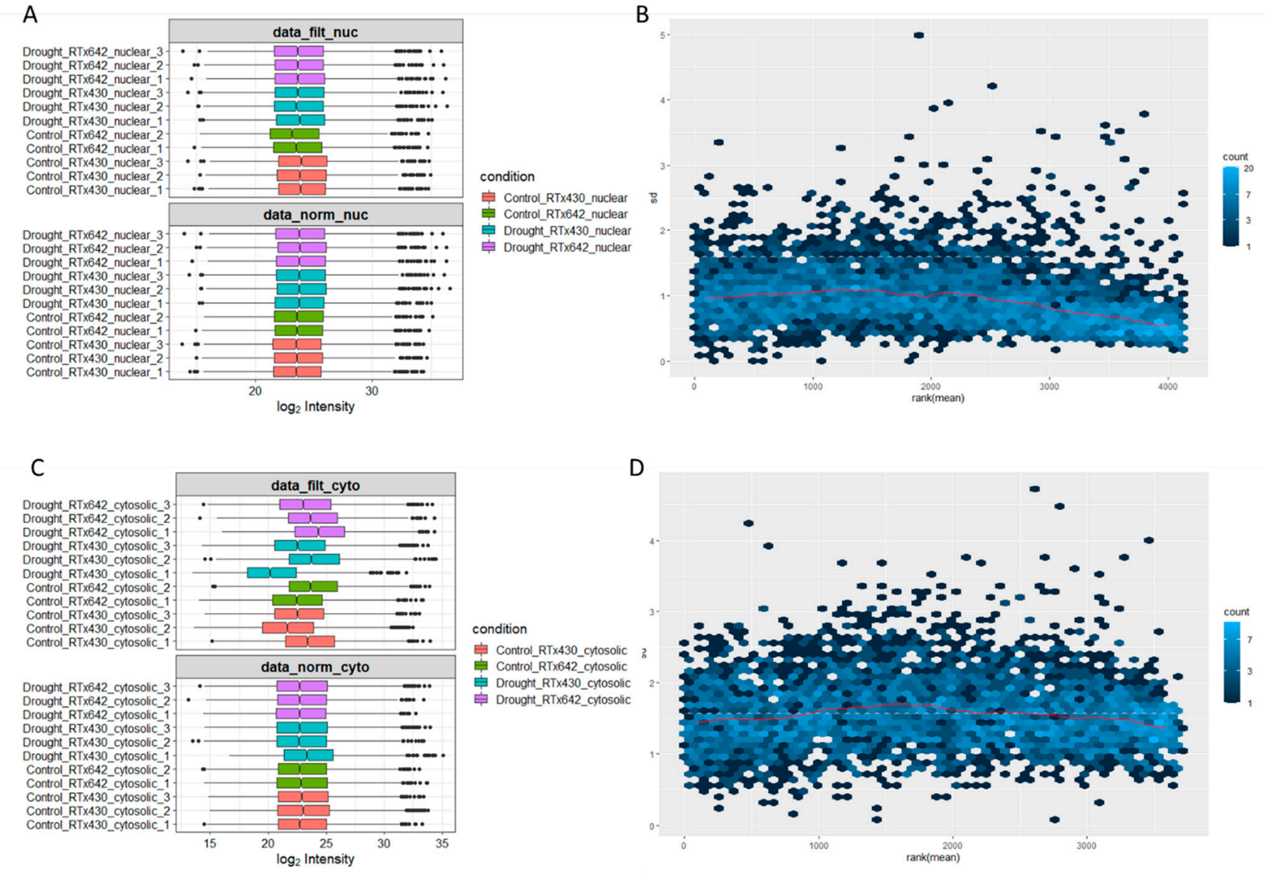

Supplemental Figure S1. Data normalization and goodness of fit. Distribution of protein abundances before and after variance-stabilized normalization (A, C), and evaluation of goodness of fit (B, D) for both organelle-enriched (top row A, B) and cytosolic-enriched samples (bottom row C, D). The horizontal red line in B and D indicate a successful variance stabilized normalization.

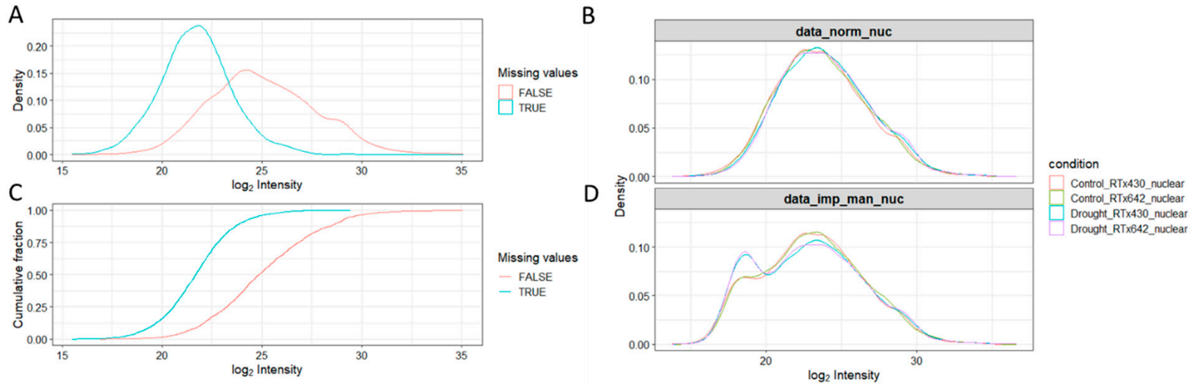

Supplemental Figure S2. Missingness caused by low abundance proteins in organelle-enriched samples. Missingness in proteomics data is often caused by proteins below the limit of detection. (A, C) The distribution of protein intensities with missing values (blue lines, “True”) and without missing values (red lines, “False”). Distribution of protein intensities pre- and post-missing value imputation (B and D, respectively).

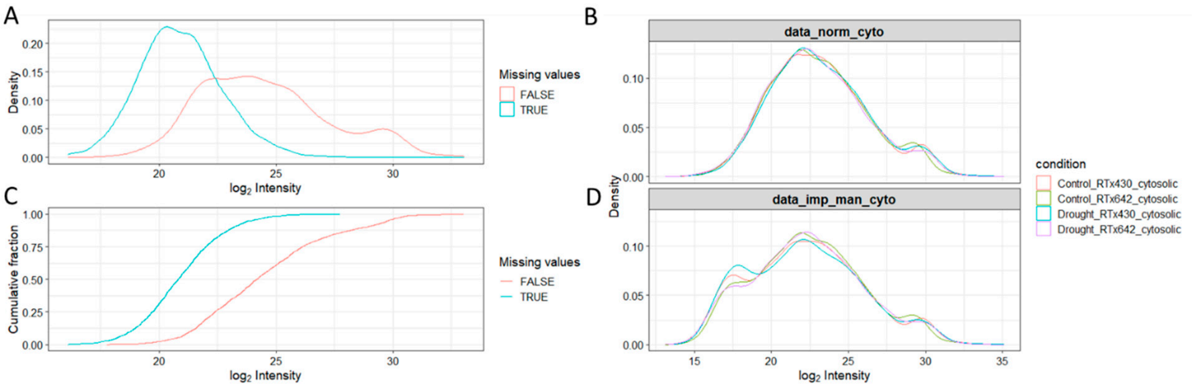

Supplemental Figure S3. Missingness caused by low abundance proteins in cytosolic-enriched samples. Missingness in proteomics data is often caused by proteins below the limit of detection. (A, C) The distribution of protein intensities with missing values (blue lines, “True”) and without missing values (red lines, “False”). Distribution of protein intensities pre- and post-missing value imputation (B and D, respectively).

References

Zhang, Xiaofei, et al. "Proteome-wide identification of ubiquitin interactions using UbIA-MS." *Nature protocols* 13.3 (2018): 530.
